# Supplementary material for: Denoising diffusion model for increased performance of detecting structural heart disease
Source: medRxiv. 2024 Nov 22:2024.11.21.24317662. Preprint. [Version 1] doi: 10.1101/2024.11.21.24317662 (PMC11601717; doi:10.1101/2024.11.21.24317662)
Supplement: 1 [file NIHPP2024.11.21.24317662v1-supplement-1.pdf]

medRxiv preprint doi: https://doi.org/10.1101/2024.11.21.24317662; this version posted November 22, 2024. The copyright holder for this preprint (which was not certified by peer review) is the author/funder, who has granted medRxiv a license to display the preprint in perpetuity.
It is made available under a CC-BY 4.0 International license .

# 10 Supplementary Material

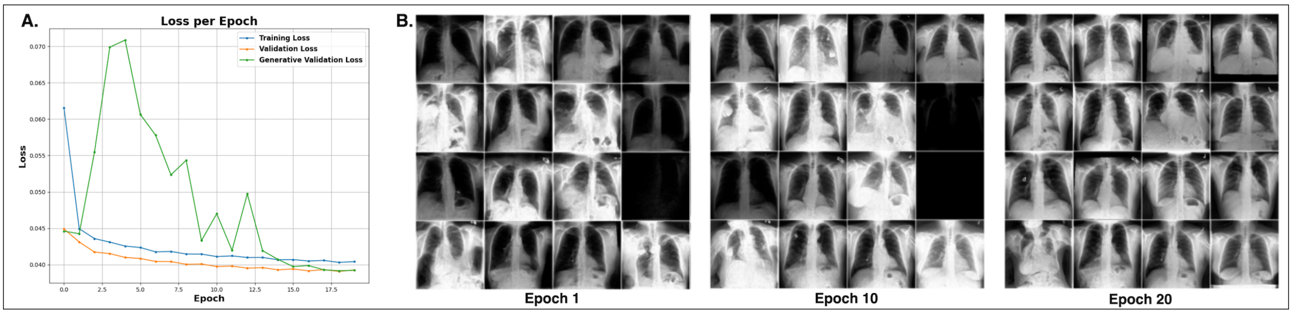


**Fig. 6 Training loss plots and evolution of generated images across epochs. a)** Depicts the training progression of the diffusion model, showing the training loss, validation loss, and generative validation loss. The training and validation losses are computed using perceptual loss, while the generative validation loss is calculated by generating a batch of images from the validation dataset and computing the MSE loss between the generated and original images. All three loss values converge and stabilize around epochs 15-20, indicating a steady state in training. **b)** Shows the progression of generated validation images across different epochs, with image quality stabilizing at a high level between epochs 15-20.

medRxiv preprint doi: https://doi.org/10.1101/2024.11.21.24317662; this version posted November 22, 2024. The copyright holder for this preprint (which was not certified by peer review) is the author/funder, who has granted medRxiv a license to display the preprint in perpetuity.
It is made available under a CC-BY 4.0 International license .

**Table 2 Diagnostic model performance across the SLVH, DLV, and composite labels.** Performance metrics for each of the four diagnostic models stratified by label, comparing brier loss, log loss, AUROC, AUPRC, precision, and specificity. Precision and specificity were both computed at a 50% recall threshold.

| | Brier loss | Log loss | AUROC | AUPRC | Precision | Specificity |
|---|---|---|---|---|---|---|
| **SLVH** | | | | | | |
| Base | 0.106 [0.098 - 0.113] | 0.343 [0.324 - 0.363] | 0.731 [0.706 - 0.760] | 0.196 [0.159 - 0.223] | 19.8% | 77.4% |
| Gen | 0.115 [0.110 - 0.120] | 0.375 [0.360 - 0.393] | 0.705 [0.681 - 0.735] | 0.217 [0.185 - 0.242] | 19.4% | 76.8% |
| Base+Gen | 0.106 [0.098 - 0.112] | 0.341 [0.325 - 0.362] | 0.730 [0.709 - 0.760] | 0.205 [0.178 - 0.233] | 20.4% | 78.2% |
| Base+Gen (Positive) | 0.103 [0.098 - 0.109] | 0.331 [0.306 - 0.350] | 0.745 [0.722 - 0.769] | 0.206 [0.172 - 0.234] | 21.4% | 79.5% |
| **DLV** | | | | | | |
| Base | 0.057 [0.052 - 0.064] | 0.209 [0.191 - 0.227] | 0.819 [0.794 - 0.841] | 0.353 [0.286 - 0.400] | 28.8% | 90.3% |
| Gen | 0.070 [0.066 - 0.076] | 0.244 [0.228 - 0.256] | 0.795 [0.770 - 0.825] | 0.276 [0.210 - 0.334] | 22.0% | 86.1% |
| Base+Gen | 0.058 [0.053 - 0.065] | 0.212 [0.193 - 0.229] | 0.817 [0.793 - 0.842] | 0.323 [0.260 - 0.371] | 27.5% | 89.7% |
| Base+Gen (Positive) | 0.058 [0.051 - 0.064] | 0.213 [0.190 - 0.231] | 0.824 [0.801 - 0.847] | 0.340 [0.253 - 0.391] | 27.8% | 89.8% |
| **Composite** | | | | | | |
| Base | 0.125 [0.117 - 0.134] | 0.396 [0.380 - 0.414] | 0.780 [0.759 - 0.797] | 0.445 [0.408 - 0.483] | 39.7% | 84.7% |
| Gen | 0.150 [0.142 - 0.155] | 0.461 [0.442 - 0.475] | 0.754 [0.735 - 0.773] | 0.419 [0.382 - 0.457] | 37.3% | 83.1% |
| Base+Gen | 0.124 [0.118 - 0.131] | 0.393 [0.369 - 0.411] | 0.786 [0.768 - 0.806] | 0.455 [0.418 - 0.491] | 40.7% | 85.4% |
| Base+Gen (Positive) | 0.119 [0.110 - 0.126] | 0.379 [0.357 - 0.405] | 0.798 [0.779 - 0.818] | 0.477 [0.441 - 0.527] | 43.1% | 86.7% |

medRxiv preprint doi: https://doi.org/10.1101/2024.11.21.24317662; this version posted November 22, 2024. The copyright holder for this preprint (which was not certified by peer review) is the author/funder, who has granted medRxiv a license to display the preprint in perpetuity.
It is made available under a CC-BY 4.0 International license .

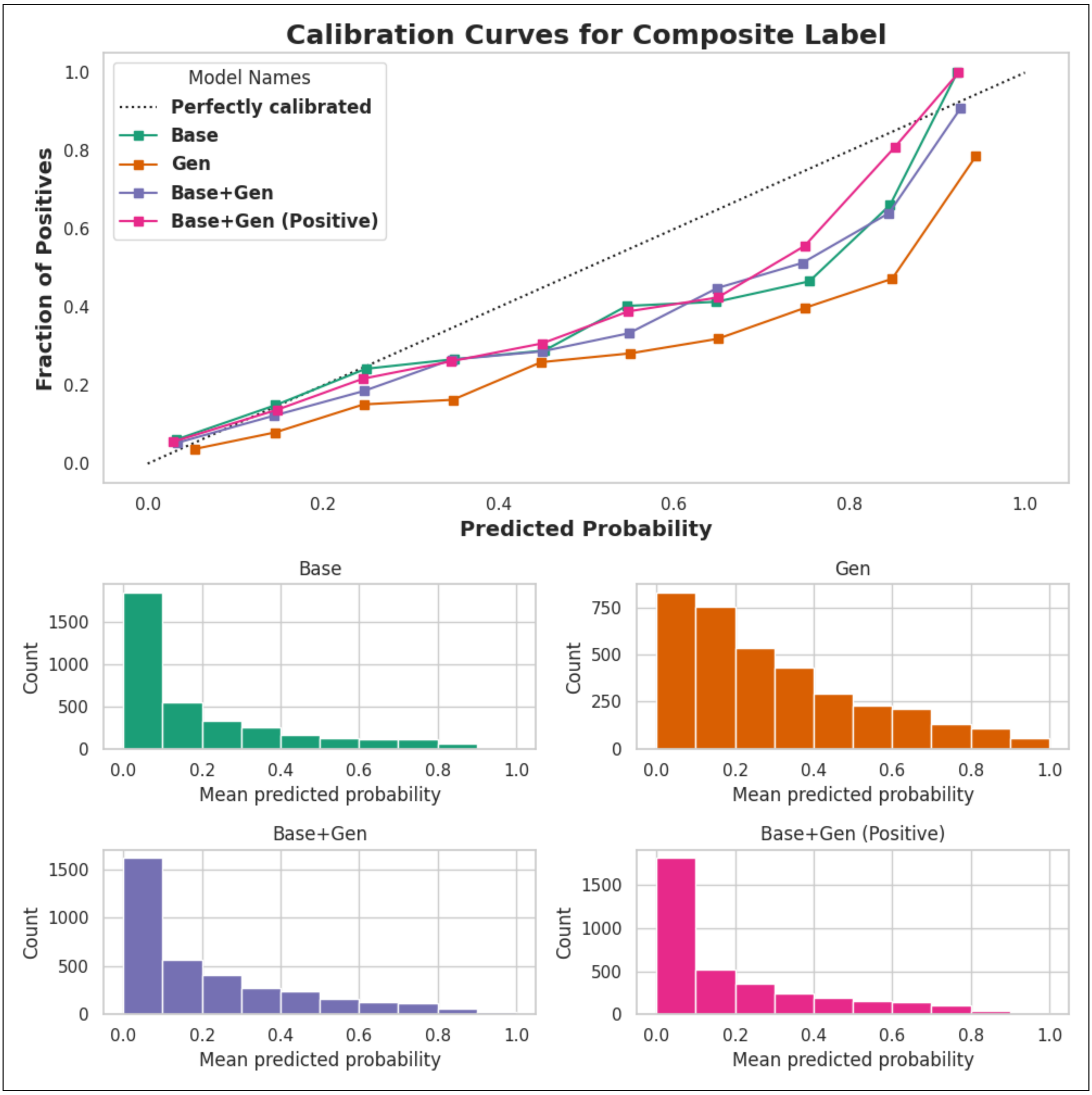


**Fig. 7 Calibration curves for the composite label across the four diagnostic models.** The calibration plot compares the predicted probabilities of positive cases for the composite label against the fraction of positive results, with the dotted line representing a perfectly calibrated model. The graph shows calibration results for the four different models of Base, Gen, Base+Gen, and Base+Gen (Positive). The Base+Gen (Positive) model demonstrates the closest alignment to the optimal calibration, indicating more confident predictions. In contrast, the Gen model shows underconfident predictions more focused in the higher probability ranges. The histogram plots show the distribution of predicted probabilities for each model.

medRxiv preprint doi: https://doi.org/10.1101/2024.11.21.24317662; this version posted November 22, 2024. The copyright holder for this preprint (which was not certified by peer review) is the author/funder, who has granted medRxiv a license to display the preprint in perpetuity.
It is made available under a CC-BY 4.0 International license .

**Table 3 Complete demographic and inference results for cross-matched images.** This table provides more detail for the images observed in Figure 2 including demographic data and inference results for each of the four diagnostic models for the provided label. The inference results show the mean-average probabilities generated by the diagnostic models for the Base, Gen, Base+Gen, and Base+Gen(Pos) datasets. While the Gen model tends to produce overly optimistic probabilities, the combined dataset models produce more balanced probabilities, effectively integrating characteristics of both the Gen and Base models.

| | Demographics | | Measurements | | | Label and Inferences | | | | |
|---|---|---|---|---|---|---|---|---|---|---|
| **Image** | **Age** | **Sex** | **IVSd** | **LVPWd** | **LVIDd** | **Ground Truth** | **Base** | **Gen** | **Base+Gen** | **Base+Gen(Pos)** |
| A | 52 | M | 1.57 | 1.33 | 4.60 | SLVH | 2.2% | 39.8% | 7.6% | 7.9% |
| B | 68 | F | 1.42 | 1.36 | 4.66 | SLVH | 0.6% | 24.1% | 6.4% | 5.0% |
| C | 66 | M | 1.25 | 1.19 | 6.21 | DLV | 31.9% | 34.0% | 19.5% | 16.8% |
| D | 76 | F | 1.21 | 1.25 | 5.37 | DLV | 2.7% | 3.8% | 0.2% | 0.2% |
| E | 47 | M | 1.93 | 1.58 | 6.26 | Composite | 73.3% | 77.9% | 56.0% | 51.7% |
| F | 79 | F | 1.50 | 1.34 | 5.89 | Composite | 46.5% | 82.0% | 58.4% | 50.9% |
| G | 82 | M | 1.00 | 1.04 | 4.90 | Normal | 60.5% | 90.5% | 76.9% | 75.7% |
| H | 51 | F | 0.92 | 0.87 | 3.80 | Normal | 98.6% | 78.6% | 95.1% | 98.1% |

medRxiv preprint doi: https://doi.org/10.1101/2024.11.21.24317662; this version posted November 22, 2024. The copyright holder for this preprint (which was not certified by peer review) is the author/funder, who has granted medRxiv a license to display the preprint in perpetuity.
It is made available under a CC-BY 4.0 International license .

**Table 4 Diagnostic model performance for the composite label across different age groups.** Performance metrics for each of the four diagnostic models on the composite label stratified by age group. Reported metrics include comparing brier loss, log loss, AUROC, and AUPRC.

| | Brier loss | Log loss | AUROC | AUPRC |
|---|---|---|---|---|
| **Age $<$ 60** | | | | |
| Base | 0.170 [0.147 - 0.190] | 0.527 [0.460 - 0.587] | 0.637 [0.574 - 0.700] | 0.285 [0.193 - 0.356] |
| Gen | 0.162 [0.141 - 0.181] | 0.510 [0.450 - 0.564] | 0.618 [0.552 - 0.686] | 0.281 [0.180 - 0.349] |
| Base+Gen | 0.162 [0.139 - 0.184] | 0.517 [0.445 - 0.582] | 0.610 [0.547 - 0.676] | 0.259 [0.164 - 0.322] |
| Base+Gen (Positive) | 0.161 [0.138 - 0.181] | 0.508 [0.443 - 0.571] | 0.628 [0.562 - 0.693] | 0.286 [0.192 - 0.356] |
| **Age 60-69** | | | | |
| Base | 0.137 [0.121 - 0.152] | 0.451 [0.401 - 0.497] | 0.782 [0.746 - 0.819] | 0.569 [0.498 - 0.634] |
| Gen | 0.157 [0.143 - 0.170] | 0.484 [0.450 - 0.518] | 0.767 [0.730 - 0.806] | 0.536 [0.467 - 0.604] |
| Base+Gen | 0.138 [0.122 - 0.152] | 0.447 [0.401 - 0.492] | 0.781 [0.745 - 0.819] | 0.586 [0.519 - 0.656] |
| Base+Gen (Positive) | 0.132 [0.117 - 0.146] | 0.438 [0.386 - 0.486] | 0.793 [0.758 - 0.830] | 0.610 [0.548 - 0.674] |
| **Age 70-79** | | | | |
| Base | 0.137 [0.120 - 0.153] | 0.424 [0.373 - 0.472] | 0.762 [0.721 - 0.806] | 0.419 [0.324 - 0.507] |
| Gen | 0.143 [0.128 - 0.157] | 0.442 [0.402 - 0.478] | 0.744 [0.700 - 0.792] | 0.431 [0.341 - 0.519] |
| Base+Gen | 0.131 [0.115 - 0.146] | 0.413 [0.367 - 0.455] | 0.764 [0.718 - 0.809] | 0.433 [0.347 - 0.513] |
| Base+Gen (Positive) | 0.131 [0.114 - 0.148] | 0.409 [0.365 - 0.453] | 0.773 [0.731 - 0.815] | 0.435 [0.345 - 0.518] |
| **Age 80-90** | | | | |
| Base | 0.170 [0.147 - 0.190] | 0.527 [0.460 - 0.587] | 0.637 [0.574 - 0.700] | 0.285 [0.193 - 0.356] |
| Gen | 0.162 [0.141 - 0.181] | 0.510 [0.450 - 0.564] | 0.618 [0.552 - 0.686] | 0.281 [0.180 - 0.349] |
| Base+Gen | 0.162 [0.139 - 0.184] | 0.517 [0.445 - 0.582] | 0.610 [0.547 - 0.676] | 0.259 [0.164 - 0.322] |
| Base+Gen (Positive) | 0.161 [0.138 - 0.181] | 0.508 [0.443 - 0.571] | 0.628 [0.562 - 0.693] | 0.286 [0.192 - 0.356] |

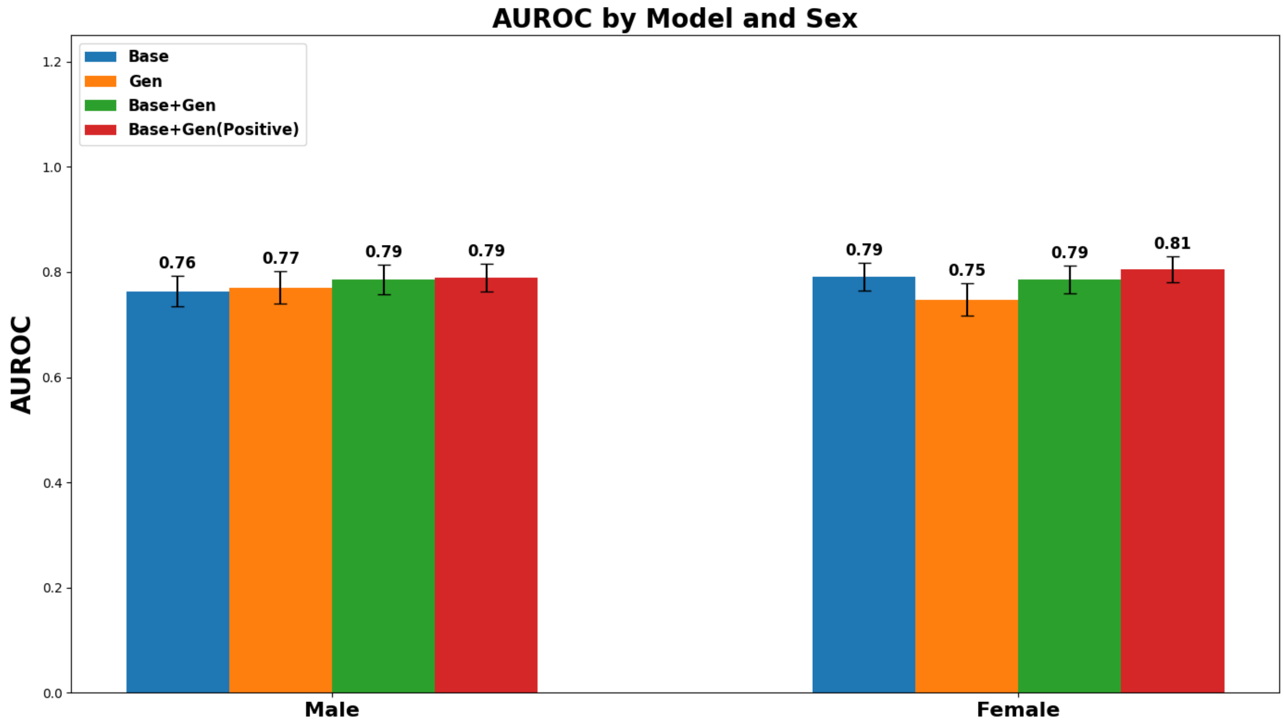


**Fig. 8 AUROC comparisons of the composite label stratified by sex for each of the diagnostic models.** Comparison of AUROC metrics for each of the four diagnostic models stratified by sex. The Gen model shows improved performance for males compared to females; however, the Base+Gen model shows equal performance across both demographics. The Base+Gen (Positive) model consistently outperforms all other models for both males and females.
